# Longitudinal Associations Between Adolescents’ mHealth App Use, Body Dissatisfaction, and Physical Self-Worth: Random Intercept Cross-Lagged Panel Study

**DOI:** 10.2196/60844

**Published:** 2025-03-11

**Authors:** Hayriye Gulec, Michal Muzik, David Smahel, Lenka Dedkova

**Affiliations:** 1 Interdisciplinary Research Team on Internet and Society Faculty of Social Studies Masaryk University Brno Czech Republic

**Keywords:** mHealth app, body dissatisfaction, physical self-worth, random intercept cross-lagged panel model, RI-CLPM, longitudinal study, adolescent

## Abstract

**Background:**

Longitudinal investigation of the association between mobile health (mHealth) app use and attitudes toward one’s body during adolescence is scarce. mHealth apps might shape adolescents’ body image perceptions by influencing their attitudes toward their bodies. Adolescents might also use mHealth apps based on how they feel and think about their bodies.

**Objective:**

This prospective study examined the longitudinal within-person associations between mHealth app use, body dissatisfaction, and physical self-worth during adolescence.

**Methods:**

The data were gathered from a nationally representative sample of Czech adolescents aged between 11 and 16 years (N=2500; n=1250, 50% girls; mean age 13.43, SD 1.69 years) in 3 waves with 6-month intervals. Participants completed online questionnaires assessing their mHealth app use, physical self-worth, and body dissatisfaction at each wave. The mHealth app use was determined by the frequency of using sports, weight management, and nutritional intake apps. Physical self-worth was assessed using the physical self-worth subscale of the Physical Self Inventory-Short Form. Body dissatisfaction was measured with the items from the body dissatisfaction subscale of the Eating Disorder Inventory-3. The random intercept cross-lagged panel model examined longitudinal within-person associations between the variables. A multigroup design was used to compare genders. Due to the missing values, the final analyses used data from 2232 adolescents (n=1089, 48.8% girls; mean age 13.43, SD 1.69 years).

**Results:**

The results revealed a positive within-person effect of mHealth app use on the physical self-worth of girls: increased mHealth app use predicted higher physical self-worth 6 months later (β=.199, *P*=.04). However, this effect was not consistent from the 6th to the 12th month: a within-person increase in using apps in the 6th month did not predict changes in girls’ physical self-worth in the 12th month (β=.161, *P*=.07). Regardless of gender, the within-person changes in the frequency of using apps did not influence adolescents’ body dissatisfaction. In addition, neither body dissatisfaction nor physical self-worth predicted app use frequency at the within-person level.

**Conclusions:**

This study highlighted that within-person changes in using mHealth apps were differentially associated with adolescents’ body-related attitudes. While increased use of mHealth apps did not influence body dissatisfaction across genders, it significantly predicted higher physical self-worth in adolescent girls 6 months later. A similar association was not observed among boys after 6 months. These findings indicate that using mHealth apps is unlikely to have a detrimental impact on adolescents’ body dissatisfaction and physical self-worth; instead, they may have a positive influence, particularly in boosting the physical self-worth of adolescent girls.

## Introduction

### Background

With the advances in digital technologies, tracking personal health data through mobile health (mHealth) apps has facilitated their use for health management [1]. mHealth apps commonly target modifiable lifestyle behaviors related to physical activity, nutrition, and weight [2]. They provide users with information about calories consumed or burned, allow for setting weight and physical activity goals, give feedback and motivation for progress, guide them through educational material on achieving goals, and facilitate connections with other users within the app [3-6].

Adolescents use mHealth apps frequently to manage their health. In a nationally representative survey of teenagers and young adults (N=1300), 49% of adolescents aged between 14 and 17 years reported using mobile apps related to health in 2018 in the United States [7]. In a subsequent nationally representative survey (N=1513), the percentage that reported using health apps for the same age group rose to 65% in 2020 [8]. More than half (N=2455, 58.2%) of the Czech adolescents aged between 11 and 16 years were using mHealth apps in 2021 [9]. In a Finnish sample, around half (N=4467, 52.6%) of adolescents aged between 11 and 15 years reported owning apps to monitor physical activity in 2016 [10]. mHealth apps are intended to promote health [11,12], and previous research has mainly examined the links between mHealth app use and physical health. Systematic and meta-analytical reviews of randomized controlled trials demonstrated the efficacy of mHealth apps in promoting adolescents’ physical activity [13,14], dietary intake [3], and weight management [15].

Recording, measuring, and monitoring one’s physical activities; dietary behaviors; and body weight via apps quantifies the body and the self in numbers that might shape how their users might think, feel, and evaluate themselves [16-19]. Specifically, self-tracking of the body through the sports activities engaged, calories consumed or burned, and weight loss achieved or failed might shape users’ attitudes toward their bodies [20]. Furthermore, this relationship could operate reciprocally because individuals’ attitudes toward their bodies may similarly influence the adoption and use of mHealth apps [21]. These connections should be particularly pronounced among adolescent users because adolescence represents a specifically sensitive period for body image and physical identity development [22], and adolescents are avid users of digital technologies [23,24]. However, the knowledge of the interplay between the use of mHealth apps and attitudes toward one’s body during adolescence is far from satisfactory, and there is practically no research examining these bidirectional associations longitudinally.

To address this gap, we used the longitudinal data of a representative sample of Czech adolescents and examined the bidirectional associations between adolescents’ physical self-worth, body dissatisfaction, and mHealth app use. In the analysis, we newly separated within-person effects from between-person effects, using the random intercept cross-lagged panel model (RI-CLPM) as an analytical approach [25,26]. Using this model, we disentangled how situational within-person changes in using mHealth apps corresponded with fluctuations in the same individual’s physical self-worth and body dissatisfaction over time and vice versa. Such research allows for the causal interpretation of observed associations between the constructs. Furthermore, it extends the limited nature of results obtained from cross-sectional studies, which describe only between-person associations.

### mHealth App Use and Physical Self-Worth

Adolescence is a critical period for physical identity development [27]. While developing a sense of identity, adolescents evaluate how they view their physicality, including physical condition, physical strength, physical attractiveness, and athletic competence [28]. An overall evaluation of these qualities defines one’s physical self-worth, which is concerned with satisfaction, confidence, and pride in one’s physical attributes and capabilities [29-32].

Research indicates an association between adolescents’ physical self-perceptions and physical activity engagement. A systematic and meta-analytical review of the studies conducted on 24,546 girls and 15,215 boys aged between 4 and 20 years revealed that children and adolescents with a more positive physical self-concept were likelier to engage in physical activity [33]. In a 4-year longitudinal study with British preadolescent boys aged between 4 and 9 years (N=531), perceived competence in sports was significantly associated with objectively measured physical activity [34]. Perceived physical activity competence predicted reduced sedentary behaviors and increased physical activity engagement 6 years later in a sample of Finnish adolescents (N=333; mean age 12.41 years) [35]. Physical self-perceptions were also associated with dieting behaviors, body image discrepancy, and social physique anxiety. For instance, in 1 study with Canadian girls aged between 15 and 16 years (N=631), physical self-perceptions significantly predicted changes in physical activity, dietary restraint, and physique anxiety 1 year later [36]. In another study, Turkish adolescents with lower global physical self-worth were more likely to report higher social physique anxiety [37]. Physical self-worth was negatively related to body image discrepancy for both genders in a large sample of American adolescents (N=5147) [38]. These findings underscore that physical self-perceptions are essential to understanding adolescents’ health-related behaviors and body image.

Considering the link between positive attitudes toward the physical self and health-related behaviors during adolescence, it is reasonable to assume that mHealth apps could appeal more to adolescents with higher physical self-worth, who are inherently more inclined to promote and manage their health [39]. This assumption suggests that positive attitudes toward physical capabilities may enhance mHealth app use. Simultaneously, the nature of this association might be reciprocal. mHealth apps are ubiquitous and provide several functions that facilitate guidance on achieving health behavior goals, increase motivation for sustained use, and offer digital companionship and social networking in the physical domain [3,5]. Therefore, it is equally plausible to assume that mere engagement and interaction with mHealth apps may provide a platform for adolescents to focus, appreciate, and trust their physical qualities, positively influencing their physical self-worth. It is noteworthy that no previous research has addressed the impact of mHealth apps on adolescents’ physical self-worth or how it is influenced by mHealth app use using a longitudinal study design.

### mHealth App Use and Body Dissatisfaction

Defined by a perceived discrepancy between ideal and actual body appearance, body dissatisfaction is a significant component of negative body image, and it refers to the subjective negative evaluation of one’s body against a socioculturally defined standard for a normative body [40]. Unlike physical self-worth, which focuses on the overall assessment of physical qualities related to the body’s functionality, body dissatisfaction involves a preoccupation with body appearance, including its size, shape, and weight [41,42]. It entails an evaluative component based on the discrepancy between the subjective appraisal of one’s body appearance and an ideal appearance [41,42]. Body dissatisfaction was associated with health-related behaviors and identified as a significant risk factor for nutritional deficiencies, disordered eating patterns, and an increased risk of eating disorders among an ethnically and socioeconomically diverse sample of American adolescents (N=2516) in a 5-year longitudinal study [43]. In addition, reviews of the literature indicated that adolescents with a negative body image were less likely to engage in physical activity [44] and more likely to report body image concerns as barriers to physical activity engagement [45]. A recent systematic review also highlighted that physical activity engagement could protect against body image–related concerns in this period [46].

Thus far, few studies have examined the association between app use and body dissatisfaction. A survey study of 643 Chinese adult users of a fitness app (WeRun) found that using the app for weight loss, diet, and exercise was negatively associated with body dissatisfaction [20], which aligns with previous conclusions indicating a positive association between participation in physical activity and positive body image [45]. Another study was conducted on adolescents aged between 14 and 16 years (N=889) attending schools in Belgium and examined the associations between app use, BMI, and healthier eating and drinking habits [47]. The results revealed that fitness and nutrition app use was associated with higher BMI. In addition, the association between fitness or nutrition app use and higher BMI was mediated by attitudes toward eating healthy for appearance, indicating that adolescents with a higher BMI could use lifestyle apps to improve their body dissatisfaction [47]. Other studies pointed to the positive effect of mHealth apps in lowering body dissatisfaction. For instance, apps specifically designed to address body dissatisfaction were shown to be efficacious in adult female samples with an elevated risk of developing body image disorders [48,49] among university students [50] and adolescents [51].

On the other hand, there is a growing concern that mHealth apps might have adverse effects. According to some authors, apps can contribute to rigid, perfectionist, and obsessive thinking around eating and exercise behaviors and increase the risk of overvaluing weight and shape as measures of self-worth in individuals at risk of developing negative body image [52-54]. These concerns are based on research that demonstrated a connection between using calorie-tracking and physical activity apps and disordered eating behaviors in young adult samples, particularly among those who reported using the apps for weight and shape purposes [55-59]. For instance, an online survey of American college students (N=491) revealed that body dissatisfaction and female sex were direct predictors of calorie-tracking app use [55]. In another study with undergraduate and graduate students (N=10,010) in the United States, weight-related self-monitoring, including the use of apps for this purpose, was significantly associated with eating disorder–related pathology in female and male participants [56]. Compulsive exercising behaviors and eating pathology were elevated in users of activity monitoring and food intake apps among British university students (N=352) [59]. These studies suggest that self-tracking of appearance-related health behaviors, such as weight, nutrition, and sports, through mHealth apps might induce or exacerbate body dissatisfaction.

Although both positive and negative outcomes seem to be supported by the existing literature, the dynamic nature of the association between mHealth app use and body dissatisfaction remains unclear. Crucially, studies that reported an association between body dissatisfaction and app use were based on cross-sectional designs with limited potential in determining the temporal order of the observed associations [52-59]. Others focused on apps that were specifically designed to promote body satisfaction and examined app use as a predictor of behavioral change in randomized controlled trials [48-51]. Both these designs ignored the possibility that attitudes toward one’s body might not only be the outcome of using mHealth apps but also be its cause. Consequently, attitudes toward one’s body appearance could serve as a motivating factor that prompts adolescents to start using mHealth apps to change their physique.

To our knowledge, only 1 prospective study examined whether eating and weight-related concerns in adolescents predicted mHealth app use during emerging adulthood using a population-based sample [21]. Longitudinal data (N=1428) were collected from adolescents aged between 12 and 16 years in 2010 and then 8 years later when they were emerging adults. The participants completed questionnaires that evaluated their eating and weight-related concerns, including body dissatisfaction at baseline. At follow-up, they provided data on mHealth app use. After adjusting for the baseline BMI, the results revealed that body dissatisfaction during adolescence predicted dietary and physical activity app use in adulthood in men. These findings confirmed a prospective association between body dissatisfaction and mHealth app use, but only for men.

### Gender

A growing body of evidence demonstrates gender differences in body-related self-perceptions [60] and health-related behaviors, such as dieting and exercise [61,62]. A lower level of physical activity was reported among girls across intercontinental physical activity surveillance initiatives [61], and adolescent girls were more likely to overestimate their weight and be less satisfied with their bodies than adolescent boys in a systematic review [62]. In addition, girls who overestimated their weight were likelier to skip breakfast than boys [62]. Traditional sociocultural appearance ideals differ across genders, emphasizing the pursuit of thinness among women and the pursuit of muscularity among men [63]. Adolescent girls become aware of appearance ideals earlier than boys and are likelier to try to lose weight to become thinner [64]. Consistent with this, girls are more likely to be motivated to do sports activities for appearance, agility, health, and weight management [65,66]. In contrast, adolescent boys’ motivation to engage in sports is more likely related to competition and building strength and persistence [65,66]. They are more likely than girls to have higher physical self-worth [38] and to value their body functionality via sports activities [67]. In addition, higher physical activity engagement is related to body satisfaction in boys, not girls [68]. The differences between genders in appearance ideals and dieting and exercise intentions might impact how they use apps and, consequently, the use outcomes. However, currently, there is no evidence on the role of gender identity in understanding these associations.

### This Study

This prospective study examined the relationships between body dissatisfaction, physical self-worth, and mHealth app use in a nationally representative sample of Czech adolescents using a 3-wave longitudinal panel design with 6-month intervals between the assessment points. We explored gender differences by modeling the associations for boys and girls separately. The analyses included age and BMI as confounders because they play an essential role in body dissatisfaction [41,69] and physical self-worth [36,70,71] during adolescence.

**Figure 1:**
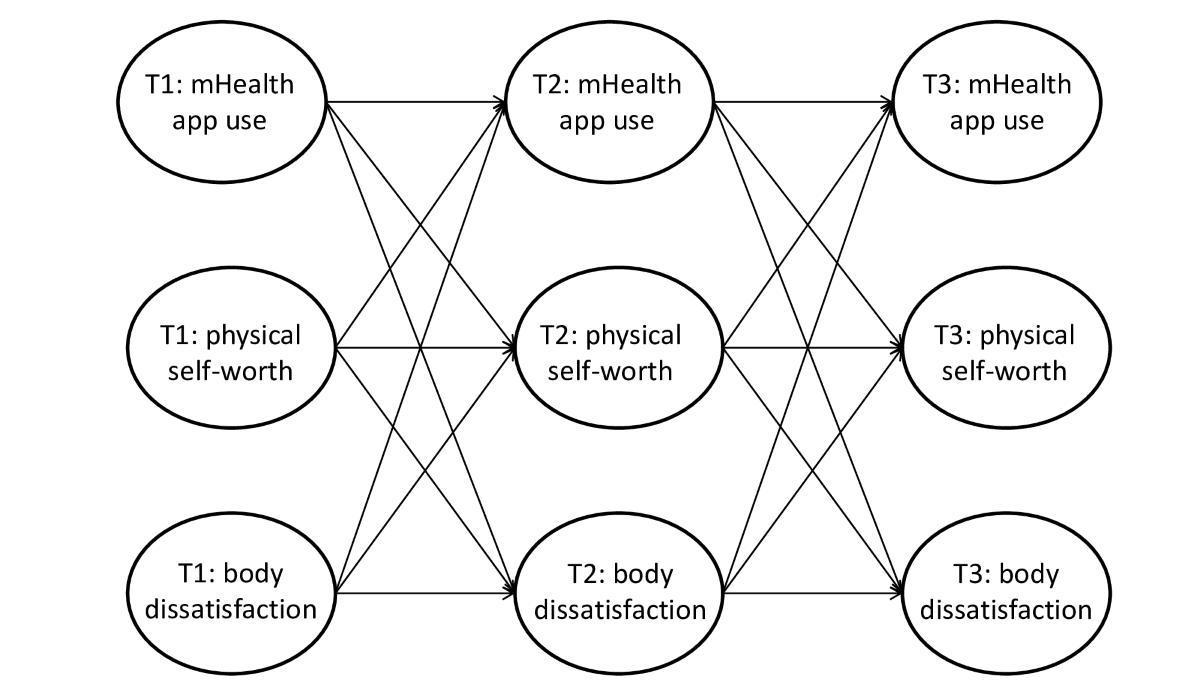
The proposed model examining within-person associations between body dissatisfaction, physical self-worth, and mobile health (mHealth) app use. T1: time 1; T2: time 2; T3: time 3.

The proposed model is displayed in Figure 1. Given the paucity of prospective research, the research aims were exploratory. We examined whether changes in the mHealth app use of an adolescent resulted in changes in body dissatisfaction and physical self-worth of the same adolescent 6 months later. We also examined the reciprocal effects and determined whether individual changes in body dissatisfaction and physical self-worth influenced the mHealth app use of the same adolescent 6 months later.

## Methods

### Recruitment

This study is part of the DigiWELL Project (Research of Excellence on Digital Technologies and Wellbeing, no. CZ.02.01.01/00/22_008/0004583) which is cofinanced by the European Union. The recruitment and data collection were carried out by a professional research agency specializing in data collection methods and applying strict quality checks and controls regarding data collection. The research agency is a member of the European Society for Opinion and Market Research and follows its standards for ethical and professional conduct of research activities. The agency selected the participants from a combined pool of 3 Czech online panels (approximately 165,000 panelists who indicated their willingness to participate in online surveys) and 980 newly recruited households. The eligibility criteria involved Czech households with at least 1 adolescent (aged between 11 and 16 years) and 1 caregiver who could complete an online questionnaire. We used quotas for the region, based on the Nomenclature of Territorial Units for Statistics; the municipality size; and the household socioeconomic status, based on the highest achieved education, to reach the proportional representation for Czech households with children. Furthermore, we set quotas for adolescents’ age and gender to ensure equal distribution. We collected 3 waves of data, 6 months apart, starting in June 2021 and finishing in June 2022.

The computer-assisted web interviewing method was used. The agency emailed their panelists an invitation to the study, which included information about the survey, eligibility, and the procedure for filling out the questionnaire. The invitation contained a link to the PDF version of the adolescent questionnaire so the parent could check what the child would be asked to complete. After filling out the basic information needed for quotas and providing active consent for their child’s participation, the parents called the respective child to complete the questionnaire. They were advised to ensure the child’s privacy during the survey completion. Adolescents were also asked for active consent and told they could refuse participation without any consequences at the beginning of the questionnaire. The survey started with a brief introduction and informed adolescents that each question included “I don’t know” and “I prefer not to say” response options. After the adolescents finished the questionnaire, they were debriefed and informed that clicking “Continue” would lock their answers, and the parents could not access them.

The survey was thoroughly pretested via cognitive interviews (ie, 30 adolescents and 2 parents) and a pilot study (ie, 195 adolescent-caregiver dyads) to ensure the comprehension of the questionnaires, check the data distributions, and determine the dimensions and internal reliabilities of the scales. Following the data collection, the research agency conducted quality checks to inspect completion times and the consistency of the entries.

The sample consisted of 1654 adolescents at time 2 (T2; n=800, 48.4%; mean age 13.43 years) and 1102 adolescents at time 3 (T3; n=532, 48.3% girls; mean age 13.37 years). The retention rate was 66% in the second wave and 44% in the third wave. Due to missing values, the final analyses used data from 2232 adolescents (n=1089, 48.8% girls; mean age 13.43, SD 1.69 years).

### Ethical Considerations

 The research ethics committee of Masaryk University approved this study (EKV-2018-068). The data and the analysis scripts are shared via the Open Science Framework [72].

### Measures

#### mHealth App Use

Adolescents answered the following question to assess mHealth app use: “You can use various applications on your phone, tablet, and other devices. Do you use applications to monitor health and exercise (eg, counting steps, tracking calories, weight, sports activities, eating/drinking, stress, sleep)?” The response options included (1) “No” and (2) “Yes.” This study focused on mHealth app use related to calorie intake and expenditure, weight management, and sports activity. Therefore, the adolescents who reported using mHealth apps were asked an additional question: “Such applications can be used to monitor or record data in various areas of health. How often have you used them in the last six months in the following areas?” The response options included (1) “calorie intake or expenditure,” (2) “weight,” and (3) “sports activity (eg, exercise, running, and working out).” These apps address lifestyle behaviors commonly used among adolescents [9]. Furthermore, the targeted lifestyle behaviors in these apps (ie, sports activities engaged, calories consumed or burned, and weight loss achieved or failed) are closely connected with body size, shape, and weight, which may influence adolescents’ perceptions of their bodies and vice versa. Although other mHealth apps are also available, such as those that track vitals, heart rate, and sleep, the targeted behaviors in these apps are not directly connected with quantifying the body size, shape, or weight. Furthermore, users might engage in them due to a medical condition.

A 7-point Likert scale determined the frequency of use: (1) “never,” (2) “once,” (3) “no more than a few times,” (4) “several times a month,” (5) “several times a week,” (6), “daily,” and (7) “several times a day.” We did not distinguish between the lifestyle behaviors addressed via apps. Therefore, an average score was computed to evaluate the frequency of mHealth app use. Adolescents who responded to the first question with a “No” (ie, adolescents who did not use mHealth apps) were categorized under the “never” frequency category at each wave. This approach allowed us to examine fluctuations in mHealth app use over time for all participants. The internal consistencies were acceptable across waves, and the Cronbach α values ranged between 0.87 and 0.88. The results supported the multigroup metric invariance over time (difference between the configural and the metric model: Δ*χ*²_6_=4.0179; *P*=.67).

#### Physical Self-Worth

Physical self-worth was measured with the physical self-worth subscale of the Physical Self Inventory-Short Form for adolescents [73,74]. It is an adapted version of the Physical Self-perception Profile developed for adults by Fox and Corbin [28]. The items were as follows: “I am proud of what I can do physically,” “I am happy with who I am and what I can do physically,” and “I am confident about my physical self-worth.” The scale applies a 6-point Likert scale (“not at all” to “entirely”). The response scale in this study included (1) “very untrue,” (2) “somewhat untrue,” (3) “neutral,” (4) “somewhat true,” and (5) “very true.” Higher scores on the scale reflected higher global physical self-worth. The internal consistencies were acceptable, and Cronbach α values ranged between 0.91 and 0.92 across the waves. The results supported the multigroup metric invariance over time (difference between the configural and the metric model: Δ*χ*^2^_6_=8.491; *P*=.20).

#### Body Dissatisfaction

Body dissatisfaction was measured with items from the body dissatisfaction subscale of the Eating Disorder Inventory-3 [75]. The original subscale consists of 10 items that evaluate dissatisfaction with the overall body shape and body parts. The scale applies a 6-point Likert scale (“never” to “always”) and measures the extent to which positive and negative evaluations relate to each body part and the overall figure. In this study, adolescents reported how satisfied versus dissatisfied they were with their overall figure, thighs, stomach, hips, and buttocks with the following response options: (1) “very dissatisfied,” (2) “rather dissatisfied,” (3) “neutral,” (4) “rather satisfied,” and (5) “very satisfied.” The items were recoded so that higher scores indicated greater body dissatisfaction. The internal consistencies were acceptable, and Cronbach α values ranged between 0.91 and 0.94 across the waves. The results supported the multigroup metric invariance over time (difference between the configural and the metric model: Δ*χ*^2^_10_=14.547; *P*=.15).

#### Age, Gender, and BMI

Adolescents indicated their age and gender at baseline. They also reported their height (in centimeters) and weight (in kilograms) to calculate their BMI. The mean BMI was 20.57 (SD 4.46; N=2220) kg/m^2^.

### Statistical Analysis

We analyzed the longitudinal data using multigroup structural equation modeling in lavaan 0.6 to 11 with the robust maximum likelihood estimator. The groups compared in the analyses were boys and girls, and we tested for differences in path estimates for these groups with likelihood ratio test. Given that we were interested in causal research questions, we tested the RI-CLPM, which decomposes observed variance into within-person and between-person levels [76]. This level of analysis accounts for the broad between-person individual differences and captures the within-person situational changes.

The model was recursive and had 2232 observations (ie, n=1143, 51.2% for boys and n=1089, 48.8% for girls) and 138 free parameters. No latent variables with multiple indicators were specified. Apart from the variables of interest, we included age and BMI, measured at wave 1, as time-invariant covariates in the model. We used full information maximum likelihood to handle missing data. This method of estimation uses all available data, including partially missing data, to produce parameter estimates, and it is an adequate method even when attrition across waves is present [77]. The attrition analysis was conducted using the multivariate ANOVA. It demonstrated that participants participating in all the waves did not differ from dropouts in the demographic and tested variables of interest (Pillai V=0.06; *F*_12,4182_=1.08; *P*=.37).

## Results

### Overview

Descriptive findings are presented in Table 1, and the intercorrelations between the variables across waves are presented in Table 2. The highest correlations were usually between the same variables measured in different waves. There were also relatively high negative correlations between physical self-worth and body dissatisfaction.

**Table 1:** The sample sizes, means, and SDs of variables across waves.

Variables	Participant sample size, n (%)	Values, mean (SD)
mHealth^a^ app use (T1)^b^	2462 (98.48)	2.29 (1.53)
mHealth app use (T2)^c^	1643 (65.72)	2.39 (1.56)
mHealth app use (T3)^d^	1091 (43.64)	2.41 (1.52)
Physical self-worth (T1)	2474 (98.96)	3.44 (1.01)
Physical self-worth (T2)	1646 (65.84)	3.46 (1.00)
Physical self-worth (T3)	1099 (43.96)	3.51 (0.98)
Body dissatisfaction (T1)	2384 (95.36)	3.45 (0.97)
Body dissatisfaction (T2)	1611 (64.44)	3.54 (0.94)
Body dissatisfaction (T3)	1073 (42.92)	3.51 (0.96)
BMI (kg/m^2^)	2232 (89.28)	20.58 (4.50)
Age^e^ (y)	2500 (100)	13.43 (1.70)

^a^mHealth: mobile health.

^b^T1: time 1.

^c^T2: time 2.

^d^T3: time 3.

^e^BMI and age are measured at time 1.

**Table 2:** Intercorrelations between variables across waves.

Variables		App use^a^ (T1)^b^	App use (T2)^c^	App use (T3)^d^	PSW^e^ (T1)	PSW (T2)	PSW (T3)	BD^f^ (T1)	BD (T2)	BD (T3)	BMI (kg/m^2^)	Age (years)
**App use (T1)**												
	*r*	1	0.535	0.469	0.120	0.123	0.078	0.011	−0.002	0.020	0.070	0.179
	*P* value	—^g^	<.001	<.001	<.001	<.001	.01	.60	.95	.52	.001	<.001
**App use (T2)**												
	*r*	0.535	1	0.520	0.124	0.172	0.139	−0.006	−0.013	0.024	0.063	0.156
	*P* value	<.001	—	<.001	<.001	<.001	<.001	.82	.59	.43	.02	<.001
**App use (T3)**												
	*r*	0.469	0.520	1	0.041	0.074	0.094	0.071	0.044	0.059	0.064	0.116
	*P* value	<.001	<.001	—	.18	.02	.002	.02	.15	.06	.04	<.001
**PSW** **(T1)**												
	*r*	0.120	0.124	0.041	1	0.606	0.566	−0.620	−0.454	−0.452	−0.193	−0.028
	*P* value	<.001	<.001	.18	—	<.001	<.001	<.001	<.001	<.001	<.001	.16
**PSW (T2)**												
	*r*	0.123	0.172	0.074	0.606	1	0.651	−0.461	−0.627	−0.537	−0.163	−0.031
	*P* value	<.001	<.001	.015	<.001	—	<.001	<.001	<.001	<.001	<.001	.21
**PSW (T3)**												
	*r*	0.078	0.139	0.094	0.566	0.651	1	−0.450	−0.527	−0.636	−0.174	−0.041
	*P* value	.01	<.001	.02	<.001	<.001	—	<.001	<.001	<.001	<.001	.21
**BD (T1)**												
	*r*	0.011	−0.006	0.071	−0.620	−0.461	−0.450	1	0.612	0.603	0.318	0.027
	*P* value	.6	.82	.02	<.001	<.001	<.001	—	<.001	<.001	<.001	.19
**BD (T2)**												
	*r*	−0.002	−0.013	0.044	−0.454	−0.627	−0.527	0.612	1	0.655	0.248	0.040
	*P* value	.95	.82	.02	<.001	<.001	<.001	<.001	—	<.001	<.001	.11
**BD (T3)**												
	*r*	0.020	0.024	0.059	−0.452	−0.537	−0.636	0.603	0.655	1	0.257	0.045
	*P* value	.52	.43	.06	<.001	<.001	<.001	<.001	<.001	—	<.001	.14
**BMI** **(kg/m** ^**2**^ **)**												
	*r*	0.070	0.063	0.064	−0.193	−0.163	−0.174	0.318	0.248	0.257	1	0.190
	*P* value	.001	.02	.04	<.001	<.001	<.001	<.001	<.001	<.001	—	<.001
**Age (years)**												
	*r*	0.179	0.156	0.116	−0.028	−0.031	−0.041	0.027	0.040	0.045	0.190	1
	*P* value	<.001	<.001	<.001	.16	.21	.18	.19	.11	.14	<.001	—

^a^Frequency of using mobile health apps.

^b^T1: time 1.

^c^T2: time 2.

^d^T3: time 3.

^e^PSW: physical self-worth.

^f^BD: body dissatisfaction.

^g^Not applicable.

The fit indices indicated an acceptable fit for the RI-CLPM, χ^2^_30_=40.99 (*P*<.001), root mean square error of approximation=0.02 (90% CI 0.00-0.03), comparative fit index=0.99, standardized root mean square residual =0.02. According to the study by Browne and Cudeck [78], root mean square error of approximation values <0.08 are reasonable for using a model. Other fit indices also indicated acceptable model data correspondence.

**Figure 2:**
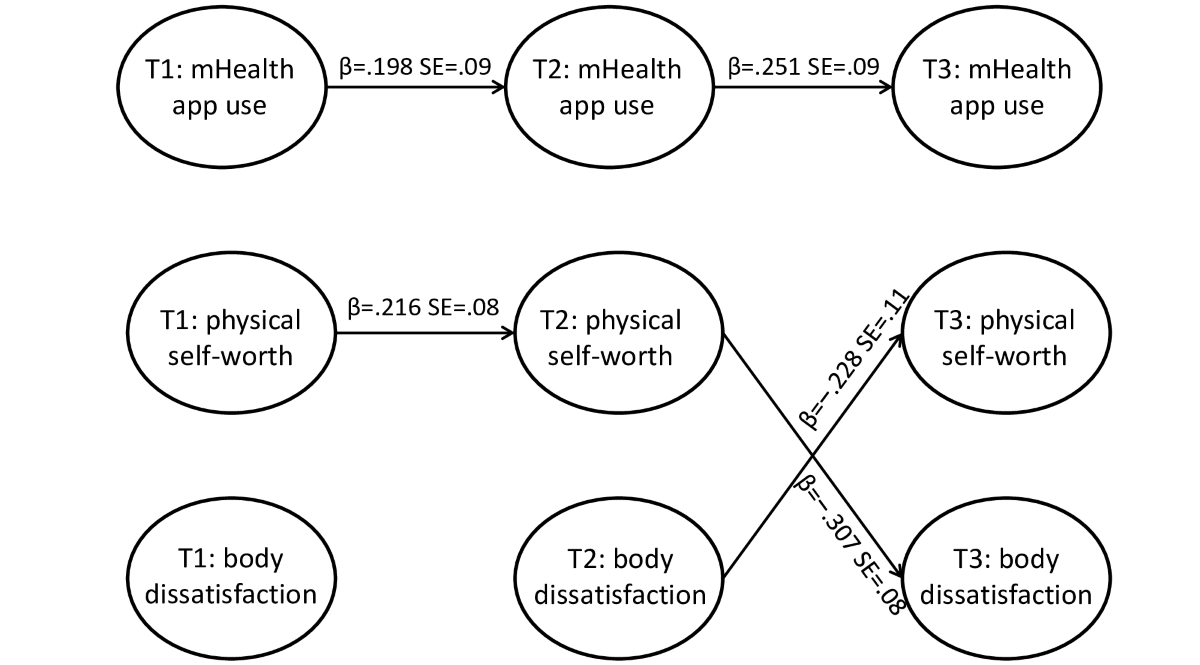
Results of the random intercept cross-lagged panel model (RI-CLPM) for adolescent boys. T1: time 1; T2: time 2; T3: time 3; SE: standard error.

**Figure 3:**
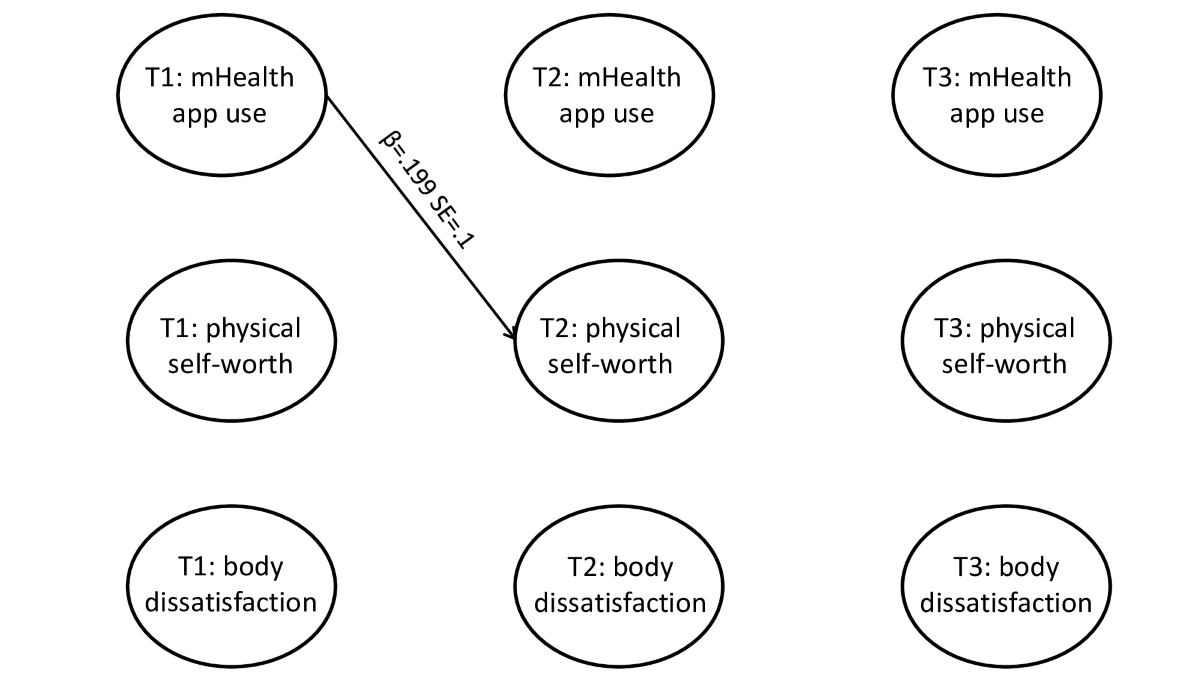
Results of the random intercept cross-lagged panel model (RI-CLPM) for adolescent girls. T1: time 1; T2: time 2; T3: time 3; SE: standard error.

The results of the RI-CLPM for boys are presented in Figure 2 and for girls in Figure 3. The figures represent only the significant paths in the analyses. For more detailed results, including the control variables and the standardized and unstandardized results of all paths, refer to Multimedia Appendix 1. The random intercepts in the RI-CLPM capture the trait-like stability; thus, we can expect lower autoregressive coefficients in general as they represent carryover effects. Our findings showed at least some carryover effect for the constructs among boys (ie, mHealth app use and physical self-worth; Figure 2), but this was not true for girls (Figure 3).

### mHealth App Use and Physical Self-Worth

In the model for boys (Figure 2), none of the lagged paths from mHealth app use were significant. Thus, when an adolescent boy used mHealth apps more frequently than usual, this change did not affect his physical self-worth 6 months later. On the other hand, in the model for girls, the lagged path from mHealth app use to physical self-worth was significant from T1 to T2 (β=.199, *P*=.04; Figure 3) but not from T2 to T3 (β=.161, *P*=.07; Table S1 in Multimedia Appendix 1), showing an inconsistency across the waves. These results show that when an adolescent girl used mHealth apps more frequently than usual, she would report higher physical self-worth 6 months later. However, an adolescent girl who reported more frequent use of mHealth apps than usual at T2 did not report higher physical self-worth at T3.

Regardless of gender, none of the lagged paths from physical self-worth were significant. Thus, when adolescents reported higher physical self-worth than usual, this change did not affect their app use frequency 6 months later.

### mHealth App Use and Body Dissatisfaction

None of the within-person associations between mHealth app use and body dissatisfaction were significant. Regardless of gender, when adolescents reported higher body dissatisfaction than usual, this change did not affect their app use frequency 6 months later. In addition, changes in app use frequency were not associated with changes in adolescents’ body dissatisfaction 6 months later.

### Body Dissatisfaction and Physical Self-Worth

Although it was not among our research interests, we found negative reciprocal within-person associations between physical self-worth and body dissatisfaction, but only among boys from T2 to T3 (Figure 2). This finding indicated that when an adolescent boy reported higher physical self-worth than usual at T2, he would likely report lower body dissatisfaction at T3 (β=–.307, *P*<.001). Similarly, when an adolescent boy reported higher body dissatisfaction than usual at T2, he would be less likely to report physical self-worth at T3 (β=−.228, *P*=.04).

### Comparison of Effect Sizes Between Genders

We examined the significance of the intergroup differences for the causal effects that were significant either among boys or girls. Although these differences were nonsignificant, some of the effects in one group were at least twice the size of the effects of the other group. Thus, we can assume that they might still have some practical meaning but should be interpreted cautiously. It was the case for the negative reciprocal within-person effects between physical self-worth and body dissatisfaction, which were significant in boys from T2 to T3 (Figure 2). For girls, there was a considerable difference in the effect of mHealth app use on physical self-worth from T1 to T2 (Figure 3).

## Discussion

### Principal Findings

#### Overview

This study used 3-wave longitudinal panel data to examine the temporal and reciprocal associations between adolescents’ body dissatisfaction, physical self-worth, and mHealth app use over time. We applied the RI-CLPM as an analytical approach to disentangle the within-person and between-person variance to determine the directionality of the associations. We used a multigroup model for boys and girls and included BMI and age as confounding variables in the analyses. This study provided the first comprehensive information on the long-term associations between adolescents’ attitudes toward their bodies and their mHealth app use.

#### mHealth App Use and Physical Self-Worth

mHealth app use had a significant within-person effect on adolescent girls’ physical self-worth from T1 to T2. In other words, an increase in the mHealth app use frequency of an adolescent girl increased the physical self-worth of the same adolescent girl 6 months later. There is no previous research to compare to our findings. However, previous randomized controlled trials demonstrated the positive impact of mHealth apps on adolescents’ health outcomes, physical activity levels, and weight outcomes [3,13-15]. Our finding expands upon these previous studies by showing that, in addition to shaping health-related behaviors (ie, physical activity levels and weight outcomes), mHealth apps could positively influence how adolescent girls view their physical selves. Previous studies reported a connection between higher physical self-perceptions and overall well-being in adolescent populations [79,80]. Our finding suggests that using apps for sports, nutritional intake, and weight management could be a viable route to enhance adolescent girls’ physical self-perceptions that might contribute to their overall well-being. Future research might examine whether enhanced physical self-perceptions mediate between mHealth app use and health-related behavior change in adolescent girls. In addition, behavioral change techniques that positively influence the physical self-perceptions of adolescent girls upon mHealth app use warrant further research attention.

A similar association was not observed in boys at 6 months. An increase in the mHealth app use frequency of an adolescent boy did not increase the physical self-worth of the same adolescent boy 6 months later. When we tested for the significance of the effect size differences between genders, we observed that the difference in effect sizes was statistically insignificant, although it was considerably large. Therefore, we decided to interpret the gender differences. One possible explanation for increased physical self-worth in girls upon mHealth use, but not in boys, could be related to gender role expectations related to health and appearance. Societal expectations place a disproportionate burden on girls to conform to a thin and fit ideal, influencing the health discourse [81]. There is also a growing emphasis on individuals taking personal responsibility for their health, further compounding this burden [17,19]. Adolescent girls often feel pressured to conform to culturally defined health standards, and they are more inclined to use self-tracking technologies to manage their appearance [82]. Previous research identified a positive association between health-related behaviors (eg, physical activity) and self-worth via increased positive perceptions about physical appearance in adolescent girls but not boys [83]. In addition, physical conditioning, body appearance, and sports competence perceptions were similarly associated with physical self-worth in adolescent girls, implying that being thin and athletic are significant determinants of their physical self-worth [84]. It is possible that using mHealth apps empowers girls more than boys by giving them a sense of self-discipline and control over their bodies to enhance their health (physical conditioning and sports competence) and manage their weight and appearance. This empowerment may contribute to heightened levels of physical self-worth in them.

However, this effect was not maintained at the 12th month (T3). In other words, there was no significant increase in physical self-worth at T3 for the adolescent girls who used mHealth apps more frequently at T2. Our findings indicate that the observed positive effect of mHealth apps on the physical self-worth of adolescent girls is not stable, and the circumstances we were unable to capture in this study might better explain the observed association. This study’s first and second waves of data collection coincided with a period when strict lockdown regulations for the COVID-19 pandemic were gradually loosening, although they were not entirely lifted. In contrast, the third wave occurred when the COVID-19 pandemic–related regulations were no longer in force. Research suggests that during the COVID-19 pandemic, adolescents experienced reduced physical activity, difficulty managing food intake, and weight gain [85-87]. It is plausible that adolescent girls appreciated enhancing their physical capabilities through the surveillance of mHealth apps more during the period between the T1 and T2 assessments—a period of relative isolation and restricted mobility—compared to the period between the T2 and T3 assessments when normalcy returned, and alternative methods for enhancing physical self-perceptions became available. However, these interpretations are speculative. Further research is required to understand the long-term effect of mHealth apps on adolescent girls’ physical self-worth.

#### mHealth App Use and Body Dissatisfaction

We found no significant within-person effect of mHealth app use on body dissatisfaction. In other words, regardless of gender, a within-person increase in using mHealth apps did not result in changes in body dissatisfaction 6 months later. There is no previous study using a longitudinal design to compare our findings. However, they contradict previous cross-sectional research that either reported a positive association between using mHealth apps and body satisfaction in young adults [20] or suggested that mHealth apps might induce or exacerbate body dissatisfaction [52-54]. Our findings indicated that after 6 months, adolescents’ body dissatisfaction did not significantly change upon mHealth app use. We recommend exploring time-specific changes in body dissatisfaction as a function of the short-term and long-term use of mHealth apps for future research. For instance, a daily diary study could provide valuable insights into the immediate impact of mHealth apps on body satisfaction or dissatisfaction. In addition, future research is required to examine whether the within-person effect of mHealth apps on body satisfaction or dissatisfaction might take longer than 6 months to manifest.

We also did not observe a significant effect of body dissatisfaction on mHealth app use when we examined the reverse relationship. In other words, regardless of gender, a change in body dissatisfaction did not change the frequency of using mHealth apps 6 months later in our sample. In the only prospective study conducted by Hahn et al [21], body dissatisfaction during adolescence was predictive of using mHealth apps in emerging adulthood (ie, 8 years later) in men. Our findings did not support the results of this study. Changes in body dissatisfaction did not shape the frequency of using mHealth apps within 6 months. One possible explanation for this inconsistency could be the time lag between the assessment points in the 2 studies. Although body dissatisfaction might predict mHealth app use in men from adolescence to early adulthood [21], such an association might not be revealed in shorter durations during adolescence, such as in 6 months, as in our study. However, it should be kept in mind that the findings of Hahn et al [21] cannot translate to a causal impact at the within-person level because they represent between-person associations. We did not observe a within-person relationship between mHealth app use and body dissatisfaction in a representative sample of adolescents using 6-month intervals between the assessment points. These findings suggest that within-person fluctuations in body dissatisfaction might not influence mHealth app use in the short term during adolescence. Instead, the overall between-person differences in body dissatisfaction during adolescence might have a more substantial influence in predicting mHealth app use in the long term.

#### Body Dissatisfaction and Physical Self-Worth

Although examining the interrelationships between body dissatisfaction and physical self-worth was not our direct research interest, our analyses produced some interesting findings. We observed a negative reciprocal association between body dissatisfaction and physical self-worth between the T2 and T3 assessments in boys but not in girls. According to that, adolescent boys who reported higher body dissatisfaction than usual at the 6th month were likelier to report lower physical self-worth at the 12th month and vice versa. A similar association was not observed among adolescent girls. We tested for the significance of the effect size differences between genders. Although the effect size difference between genders was statistically insignificant, it was considerably large and might have practical implications. Body dissatisfaction is a significant risk factor for the increased rates of mental disorders in general, including eating disorders in boys [88,89]. Our findings indicate that dissatisfaction with body appearance and lower esteem in physical attributes are mutually reinforced in adolescent boys, which might increase the risk for eating disorders by creating a vicious cycle. Since boys are acculturated to obtain a muscular body appearance, and lately also a lean appearance, they might be more likely than girls to focus on physical qualities (ie, what they can do physically) to evaluate body appearance (ie, muscularity), and vice versa. These findings imply that interventions that address body dissatisfaction might benefit from components that enhance the physical self-concept in boys. Improving satisfaction, confidence, and pride in physical attributes in boys could have a causal mitigating effect on subjective negative evaluations regarding their body appearance. However, it should be noted that this effect was observed only between the T2 to T3 assessments, not between the T1 to T2 assessments; thus, whether the reciprocal association takes longer to manifest in boys or whether it is pronounced after the COVID-19 pandemic situation deserves further research attention.

### Limitations and Future Research

Several limitations should be taken into account when evaluating the findings of this study. We did not examine mHealth apps separately. The decision was based on the observation that several mHealth apps for physical activity (such as Fitbit) also provide weight and calorie intake information. Thus, distinguishing between lifestyle behaviors would be arbitrary. In addition, we were interested in evaluating the most frequent lifestyle behaviors addressed via mHealth apps rather than specifically focusing on using certain apps. However, using an average score for app use instead of examining each lifestyle behavior separately (eg, weight, diet, and physical activity) might have limited our findings. Apps use diverse methods to collect data, including self-reported subjective information and objective data through embedded technologies such as GPS. We did not distinguish on the basis of the data collection type in this study and relied on subjective information regarding the frequency of using mHealth apps. Relying on subjective data limited our ability to control the response characteristics of the participants. The analyses included age and BMI as time-invariant control variables due to their robust associations with the study variables in previous literature. Although other variables, such as socioeconomic status, could also provide significant insights, a more parsimonious model was preferred given the complexity of RI-CLPM.

We assessed mHealth use based on the frequency of use and thus cannot ascertain if it resulted from health promotion efforts or unhealthy weight control behaviors. We interpreted the inconsistencies in significant findings across the waves based on the COVID-19 pandemic circumstances. However, it should be noted that our assessments coincided with Spring twice and Autumn once, which may also differentially impact time-specific individual deviations in the observed variables. Body dissatisfaction and physical self-worth measures were adaptations of frequently used questionnaires in the field, but they were not adequately validated. Nevertheless, we conducted cognitive testing with a subsample of participants to ensure the comprehensibility of items and the quality of the assessment procedure. Finally, our data did not include nonbinary or gender-diverse adolescents; thus, the findings cannot be generalized across a broader gender spectrum.

Future research can explore whether the observed associations might differ by the lifestyle behavior addressed via mHealth apps (eg, weight, diet, and physical activity) and the patterns of mHealth use (ie, functional vs dysfunctional use of mHealth apps). We also need future studies to replicate these findings in various countries and cultural backgrounds with data from gender-diverse samples of adolescents.

### Conclusions

This study is the first to examine the longitudinal reciprocal associations between adolescents’ physical self-worth, body dissatisfaction, and mHealth app use in a nationally representative sample of adolescents using a within-person approach. The findings revealed that within-person changes in body dissatisfaction and physical self-worth did not predict the frequency of using mHealth apps 6 months later. However, mHealth apps played differential roles in adolescents’ body-related attitudes at the within-person level. Although inconsistent, we found that a within-person increase in using mHealth apps predicted higher physical self-worth in girls 6 months later. A similar association was not observed among boys. Regardless of gender, increased use of mHealth apps was not associated with body dissatisfaction. These findings suggest that mHealth apps are unlikely to harm adolescents’ attitudes toward their body appearance and physical self-worth. Furthermore, the use of these apps may even contribute to enhancing physical self-worth among adolescent girls.

## Appendix

Multimedia Appendix 1 Standardized and unstandardized estimates for the random intercept cross-lagged panel model.

## Abbreviations

mHealth: mobile health
RI-CLPM: random intercept cross-lagged panel model
