# Supplementary material for: Longitudinal Associations Between Adolescents’ mHealth App Use, Body Dissatisfaction, and Physical Self-Worth: Random Intercept Cross-Lagged Panel Study
Source: JMIR Ment Health. 2025 Mar 11;12:e60844. doi: 10.2196/60844 (PMC11937715; doi:10.2196/60844)
Supplement: Multimedia Appendix 1 [file mental_v12i1e60844_app1.docx]

# Multimedia Appendix 1. Standardized and Unstandardized estimates for the RI-CLPM

Table S1. Standardized estimates for the RI-CLPM (girls)

| Regression path | | Time 1 🡪Time 2 | | | Time 2 🡪Time 3 | | |
| --- | --- | --- | --- | --- | --- | --- | --- |
|  | | Estimate [95% CI] | SE^a^ | *P* value | Estimate [95% CI] | SE | *P* value |
| **App use^b^** | |  |  |  |  |  |  |
|  | App use | .084 [-.106; .274] | .097 | .387 | -.021 [-.241; .172] | .099 | .830 |
|  | PSW^c^ | .143 [-.012; .299] | .079 | .071 | .076 [-.118; .269] | .099 | .444 |
|  | BD^d^ | -.107 [-.276; .062] | .086 | .214 | .013 [-.168; .195] | .092 | .885 |
|  | BMI^e,f^ | .035 [-.030; .101] | .033 | .290 | .036 [-.031; .103] | .034 | .291 |
|  | Age^f^ | .169 [.117; .222] | .027 | <.001^g^ | .173 [.120; .226] | .027 | <.001^g^ |
| **PSW** | |  |  |  |  |  |  |
|  | App use | .199 [.012; .387] | .096 | .037^g^ | .161 [-.014; .336] | .089 | .071 |
|  | PSW | .049 [-165; .264] | .109 | .654 | .118 [-.095; .332] | .109 | .277 |
|  | BD | .103 [-.091; .297] | .099 | .298 | -.087 [-.263; .088] | .090 | .328 |
|  | BMI | -.225 [-303; -.148] | .039 | <.001^g^ | -.221 [-.298; -.145] | .039 | <.001^g^ |
|  | Age | -.026 [-.079; .026] | .027 | .326 | -.026 [-.077; .026] | .026 | .327 |
| **BD** | |  |  |  |  |  |  |
|  | App use | -.034 [-.216; .148] | .093 | .714 | .035 [-149; .218] | .094 | .713 |
|  | PSW | .012 [-.203; .228] | .110 | .910 | -.214 [-.437; .010] | .114 | .061 |
|  | BD | -.070 [-.322; .183] | .129 | .588 | -.033 [-.241; .175] | .106 | .756 |
|  | BMI | .310 [.208; .413] | .052 | <.001^g^ | .303 [.201; .405] | .052 | <.001^g^ |
|  | Age | .000 [-.052; .053] | .027 | .987 | .000 [-.051; .052] | .026 | .987 |

^a^SE: Standard error.
^b^App use: Frequency of using mHealth apps.
^c^PSW: physical self-worth.
^d^BD: body dissatisfaction. ^e^BMI: body mass index.
^f^BMI and age were included as covariates in the analyses.
^g^Significant *P* value

Table S2. Standardized estimates for the RI-CLPM (boys)

| Regression path | | Time 1 🡪Time 2 | | | Time 2 🡪Time 3 | | |
| --- | --- | --- | --- | --- | --- | --- | --- |
|  | | Estimate [95% CI] | SE^a^ | *P* value | Estimate [95% CI] | SE | *P* value |
| **App use^b^** | |  |  |  |  |  |  |
|  | App use | .198 [.015; .381] | .093 | .034^c^ | .251 [.069; .433] | .093 | .007^c^ |
|  | PSW^d^ | .075 [-.063; .211] | .069 | .272 | .024 [-.131; .178] | .079 | .763 |
|  | BD^e^ | -.052 [-.197; .119] | .084 | .537 | -.031 [-.210; .148] | .092 | .735 |
|  | BMI^f,g^ | .062 [.014; .110] | .025 | .012^b^ | .063 [.014; .112] | .025 | .011^c^ |
|  | Age^g^ | .126 [.077; .175] | .025 | <.001^c^ | .128 [.079; .177] | .025 | <.001^c^ |
| **PSW** | |  |  |  |  |  |  |
|  | App use | .069 [-.051; .189] | .061 | .262 | .053 [-.083; .190] | .070 | .444 |
|  | PSW | .216 [.053; .378] | .083 | .009^c^ | .166 [-.039; .372] | .105 | .112 |
|  | BD | -.154 [-.321; .012] | .085 | .069 | -.228 [-.443; -.012] | .110 | .038^c^ |
|  | BMI | -.179 [-.244; -.115] | .033 | <.001^c^ | -.192 [-.261; -.124] | .035 | <.001^c^ |
|  | Age | .056 [.005; .108] | .026 | .033^c^ | .060 [.005; .116] | .028 | .033^c^ |
| **BD** | |  |  |  |  |  |  |
|  | App use | -.121 [-.277; .035] | .080 | .128 | .028 [-.098; .154] | .064 | .660 |
|  | PSW | -.107 [-.284; .070] | .090 | .237 | -.307 [-.453; -.161] | .075 | <.001^c^ |
|  | BD | .133 [-.118; .383] | .128 | .300 | .187 [.000; .353] | .095 | .050 |
|  | BMI | .326 [.250; .402] | .039 | <.001^c^ | .316 [.243; .390] | .038 | <.001^c^ |
|  | Age | -.071 [-.124; -.019] | .027 | .008^c^ | -.069 [-.121; -.018] | .026 | .008^c^ |

^a^SE: Standard error
^b^App use: Frequency of using mHealth apps.
^c^Significant *P* value
^d^PSW: physical self-worth.
^e^BD: body dissatisfaction.
^f^BMI: body mass index.
^g^BMI and age were included as covariates in the analyses.

Table S3. Unstandardized estimates for the RI-CLPM (boys)

| Regression path | | Time 1 🡪Time 2 | | | Time 2 🡪Time 3 | | |
| --- | --- | --- | --- | --- | --- | --- | --- |
|  | | Estimate [95% CI] | SE^a^ | *P* value | Estimate [95% CI] | SE | *P* value |
| **App use^b^** | |  |  |  |  |  |  |
|  | App use | .200 [.012; .388] | .096 | .037^c^ | .243 [.069; .418] | .089 | .006^c^ |
|  | PSW^d^ | .118 [-.093; .329] | .108 | .273 | .036 [-.198; .270] | .119 | .762 |
|  | BD^e^ | -.093 [-.384; .199] | .149 | .534 | -.056 [-.377; .266] | .164 | .734 |
|  | BMI^f,g^ | .023 [.005; .040] | .009 | .012^c^ | .023 [.005; .040] | .009 | .012^c^ |
|  | Age^g^ | .115 [.070; .161] | .023 | <.001^c^ | .115 [.070; .161] | .023 | <.001^c^ |
| **PSW** | |  |  |  |  |  |  |
|  | App use | .044 [-.034; .123] | .040 | .264 | .030 [-.047; .107] | .038 | .444 |
|  | PSW | .216 [.050; .383] | .085 | .011^c^ | .147 [-.034; .328] | .092 | .111 |
|  | BD | -.176 [-.357; .006] | .093 | .058 | -.238 [-.458; -.017] | .112 | .034^c^ |
|  | BMI | -.041[-.056; -.026] | .008 | <.001^c^ | -.041 [-.056; -.026] | .008 | <.001^c^ |
|  | Age | .033 [.003; .063] | .015 | .034^c^ | .033 [.003; .063] | .015 | .034^c^ |
| **BD** | |  |  |  |  |  |  |
|  | App use | -.066 [-.151; .018] | .043 | .124 | .016 [-.056; .088] | .037 | .658 |
|  | PSW | -.091 [-.239; .058] | .076 | .233 | -.275 [-.405; -.145] | .066 | <.001^c^ |
|  | BD | .128 [-.117; .372] | .125 | .305 | .198 [.002; .394] | .100 | .048^c^ |
|  | BMI | .067 [.050; .084] | .009 | <.001^c^ | .067 [.050; .084] | .009 | <.001^c^ |
|  | Age | -.037 [-.065; -.009] | .014 | .009^c^ | -.037 [-.065; -.009] | .014 | .009^c^ |

^a^SE: Standard error
^b^App use: Frequency of using mHealth apps.
^c^Significant *P* value
^d^PSW: physical self-worth.
^e^BD: body dissatisfaction.
^f^BMI: body mass index.
^g^BMI and age were included as covariates in the analyses.

Table S4. Unstandardized estimates for the RI-CLPM (girls)

| Regression path | | Time 1 🡪Time 2 | | | Time 2 🡪Time 3 | | |
| --- | --- | --- | --- | --- | --- | --- | --- |
|  | | Estimate [95% CI] | SE^a^ | *P* value | Estimate [95% CI] | SE | *P* value |
| **App use^b^** | |  |  |  |  |  |  |
|  | App use | .085 [-.110; .280] | .100 | .392 | -.020 [-.207; .166] | .095 | .830 |
|  | PSW^c^ | .241 [-.021; .503] | .134 | .071 | .128 [-.203; .459] | .169 | .448 |
|  | BD^d^ | -.209 [-.543; .125] | .170 | .220 | .025 [-.317; .367] | .175 | .885 |
|  | BMI^e,f^ | .012 [-.010; .034] | .011 | .291 | .012 [-.010; .034] | .011 | .291 |
|  | Age^f^ | .156 [.107; .204] | .025 | <.001^g^ | .156 [.107; .204] | .025 | <.001^g^ |
| **PSW** | |  |  |  |  |  |  |
|  | App use | .115 [.010; .220] | .054 | .032^g^ | .095 [-.007; .198] | .052 | .068 |
|  | PSW | .047 [-158; .252] | .105 | .656 | .124 [-.095; .343] | .112 | .268 |
|  | BD | .114 [-.100; .328] | .109 | .295 | -.102 [-.301; .098] | .102 | .318 |
|  | BMI | -.049 [-066; -.031] | .009 | <.001^g^ | -.049 [-066; -.031] | .009 | <.001^g^ |
|  | Age | -.016 [-.047; .016] | .016 | .326 | -.016 [-.047; .016] | .016 | .326 |
| **BD** | |  |  |  |  |  |  |
|  | App use | -.018 [-.112; .076] | .048 | .713 | .019 [-082; .119] | .051 | .713 |
|  | PSW | .011 [-.174; .195] | .094 | .910 | -.205 [-.411; .001] | .105 | .051 |
|  | BD | -.069 [-.319; .180] | .127 | .586 | -.035 [-.258; .188] | .114 | .758 |
|  | BMI | .066 [.043; .090] | .012 | <.001^g^ | .066 [.043; .090] | .012 | <.001^g^ |
|  | Age | .000 [-.031; .031] | .016 | .987 | .000 [-.031; .031] | .016 | .987 |

^a^SE: Standard error.
^b^App use: Frequency of using mHealth apps.
^c^PSW: physical self-worth.
^d^BD: body dissatisfaction. ^e^BMI: body mass index.
^f^BMI and age were included as covariates in the analyses.
^g^Significant *P* value
